# Editorial: Roles of the first and second messengers in reproduction

**DOI:** 10.3389/fendo.2024.1494529

**Published:** 2024-10-09

**Authors:** Xiaoning Zhang, Rujun Ma

**Affiliations:** ^1^ Institute of Reproductive Medicine, Medical School, Nantong University, Nantong, Jiangsu, China; ^2^ Department of Reproductive Medicine, Nanjing Jinling Hospital, Affiliated Hospital of Medical School, Nanjing University, Nanjing, Jiangsu, China

**Keywords:** first messenger, second messenger, sperm, oocyte, calcium signaling, infertility

Cell signaling is stimulated by the extracellular first messengers, such as ligands or cytokines, or mediated by the intracellular second messengers, like calcium ions, inositol 1,4,5-trisphosphate, cyclic adenosine monophosphate, diacylglycerol, and reactive oxygen species, and plays a crucial role in mammalian reproduction. Spermatogenesis, oogenesis, gamete function regulation, fertilization, assisted reproduction and other reproductive processes are all involved in the participation of first and second messengers. Their dysfunction could result in infertility in both men and women. Investigating the role of first and second messengers and uncovering the underlying signal transduction networks and mechanisms in the reproductive process mentioned above are very important for the diagnosis and treatment of infertility and human reproductive health.

In this Research Topic, Xiaoning Zhang, Jan Tesarik, and Rujun Ma aimed to focus on the role of first and second messengers and the underlying mechanisms in reproductive processes in mammals. The Research Topic includes three original articles and one review article on sperm motility and function regulation, placental syndromes and oocyte quality and functionality, which has facilitated the understanding of the roles of first and second messengers in male and female reproduction.

## Adenosine A2A receptor and lipidomics in sperm function

Adenosine could serve as the first messenger to activate the adenosine A2A receptor (A2AR), which subsequently leads to changes in sperm functions including motility and capacitation by stimulating intracellular cAMP synthesis and dynamin ATPase activation. Chen H. et al. first confirmed the presence of A2AR on the sperm flagellum and further found that the level of A2AR is positively correlated with progressive motility and associated with the fertilization rate of *in vitro* fertilization. Mechanistically, Chen H. et al. demonstrated that A2AR regulates sperm function by modulating Ca^2+^ influx through the CatSper channel by using the A2AR agonist (regadenoson) and the antagonist (SCH58261). Under the combined action of extracellular signal and intracellular second messenger, sperm undergo a variety of changes during capacitation and acrosome reaction. Cheng et al., characterized the lipidomic profiles in uncapacitated, capacitated, and acrosome-reacted human spermatozoa. Then, 12 lipids or metabolites were recognized differentially between A23187-induced acrosome-reacted spermatozoa and the uncapacitated/capacitated sperm in humans. Given that A23187 plays a role in regulating sperm function through calcium signaling, lipid metabolites were remarkably altered after capacitation and acrosome reactions, which suggested that there might be very complicated crosstalk between second messenger-modulated sperm function and lipid metabolism. Their findings provide novel candidates to address the regulatory mechanisms of capacitation and acrosome reactions in the future.

## First and second messengers in placentation and regulation of oocyte quality and functionality

First and second messengers are also crucial for female reproduction. Using the placental syndrome model, Huang et al., found that many first messengers, inflammatory cytokines, such as oncogene A, interleukin-1b, interleukin-9, interleukin-10, interleukin-18, macrophage migration inhibitory factor, macrophage colony-stimulating factor, hepatocyte growth factor, platelet-derived growth factor BB and TNF-related apoptosis-inducing ligand, may be causally associated with placental syndromes employing the Mendelian randomization analysis. Furthermore, based on molecular docking technology, they found that many active ingredients isolated from traditional Chinese medicine, puerarin, magnolol, atractylenolide I, paeoniflorin, tumulosic acid, and wogonin, are closely bound to these inflammatory cytokines, providing a novel direction and potential targets for the study and treatment of placental syndromes.

Among second messengers, calcium signaling has been the most extensively and deeply studied in all aspects of female reproduction. Chen C. et al., systematically reviewed the latest research progress on calcium signaling in oocyte maturation, activation, fertilization, function regulation of granulosa and cumulus cells and offspring development under physiological and pathological conditions. During oocyte maturation, ER stores, extracellular fluid and mitochondrial stores are the main sources and regulators of Ca^2+^, which affects the formation of the first polar body of oocytes, meiotic division, and the maintenance of chromosome and spindle conformation. The authors updated the key molecules and pathways involved in calcium homeostasis regulation in oocyte maturation and fertilization and the methods or alternative proposals of artificial oocyte activation proposals by modulating calcium signaling. In this review, Chen C. et al., also elucidated other regulatory molecules that indirectly affect the Ca^2+^ pump or downstream pathways. In addition to germ cells, granulosa cells (GC) and cumulus cells (CC), are modulated by calcium signaling. Therefore, Chen C. et al., for the first time summarized calcium signaling in GC and CC. Furthermore, adverse stress-induced Ca^2+^-related ovarian dysfunction due to the oocytes and GC damage is also a concern. Last but not least, considering the current state of this research, the authors proposed further research directions and suggestions on calcium signaling in female reproduction.

